# Impact of Physician Storytelling and Peer-to-Peer Conversation on Medical Students’ Coping Skills and Anticipatory Fear Surrounding Patient Death: A Pre-post Cohort Study

**DOI:** 10.7759/cureus.97184

**Published:** 2025-11-18

**Authors:** Shawn Koh, Yuu Ohno, Hira Khan

**Affiliations:** 1 Department of Medical Education, California University of Science and Medicine, Colton, USA

**Keywords:** end-of-life-care, medical student teaching, patient death, stress coping, thanatophobia, undergraduate medical student

## Abstract

Objective

Pre-clerkship medical students often struggle with anticipatory fear of experiences involving patient death. Various multi-session interventions have shown promise in alleviating these fears. In this study, we investigated the impact of a single-session intervention centered on physician storytelling and peer-to-peer discussion on coping skills and anticipatory fear surrounding patient death.

Methods

A total of 21 second-year pre-clerkship medical students participated in the study. Students participated in a 90-minute session involving pre-recorded interviews of physicians sharing experiences with patient death. During several points throughout the session, students discussed their reflections in small groups. Serial pre- and post-session surveys evaluated anticipatory fears related to patient death and self-efficacy to cope with death using the Thanatophobia Scale and Bugen’s Coping With Death Scale, respectively. Data were analyzed for significance using the paired t-test.

Results

Statistically significant improvements were noted in both the Thanatophobia Scale and Bugen’s Coping With Death Scale scores immediately after the session. Mean thanatophobia scores improved from a pre-session score of 23.9 to a post-session score of 21.5 (p = .030). Mean Bugen’s Coping With Death Scale scores also improved from a pre-session score of 38.3 to a post-session score of 43.0 (p = .003).

Conclusion

While anticipatory fears of patient death are common among pre-clerkship medical students, many medical schools may struggle to find space to address this concern in their already packed curricula. This study suggests a feasible and effective method for addressing this aspect of undergraduate medical education.

## Introduction

Previous studies have shown that many pre-clerkship medical students do not feel adequately prepared for end-of-life care or the emotions that encompass it [1]. Being a caregiver in the face of death can be challenging and anxiety-inducing, from delivering bad news to patients and their families [2] to not knowing the appropriate place to express emotions when a patient dies [1]. Some students have also felt a discrepancy in attitudes towards patient death, between students and more experienced providers, where the more experienced group trivializes death after years of training [1]. Others express a more existential anxiety towards death and the role of the physician in allowing death [3].

Studies have used a variety of scales in assessing students’ attitudes towards death. One study assessed medical students using the Thanatophobia Scale, a seven-question instrument specifically designed to evaluate anxiety and fear towards death, and found that anxiety towards death was highest in second-year medical students [4]. Another study used the Self-efficacy in Palliative Care (SEPC) tool, made specifically to address confidence in patient interactions, to determine if simulated experiences of caring for a dying patient and family can improve the confidence and preparedness of medical students to provide such care. This study found a statistically significant post-simulation increase in confidence with sustained confidence at the six-month point [5]. Bugen’s Coping With Death Scale was used by another study to evaluate if a mentoring program could increase student coping skills; it found an increase in student coping scores after the intervention [6]. Other such scales exist, including the Brief Humanizer Scale [6] and the Frommelt Attitude Toward the Care of the Dying Scale (FATCOD) [7].

Our research aimed to assess the impact of storytelling and peer discussion on pre-clerkship medical students’ anticipatory concerns towards patient death. Using a combination of Bugen’s Coping With Death Scale and the Thanatophobia Scale, we explored whether a single session of hearing physician perspectives and reflecting with peers would positively impact student attitudes toward death and self-efficacy of coping with death.

## Materials and methods

Study setting and design

This study was conducted at the California University of Science and Medicine on June 16, 2025. The session titled “Experiences With Patient Death” was delivered as a part of an eight-day boot camp elective for second-year medical students about to begin core clerkships. A single 90-minute session on physicians’ experiences with patient death was delivered to students, with scores/measurements obtained immediately prior to and following the session. This study was approved as exempt by the IRB of the California University of Science and Medicine (protocol number HS-2025-34) on June 2, 2025. Investigators included the director of the boot camp course and two clerkship medical students.

Participant selection

Student participants chose to enroll in this elective course prior to beginning clinical rotations. Of the 101 students in this phase of the MD program, 25 students enrolled in this course, and 23 students attended (22 in person, 1 virtually) the session on “Experiences With Patient Death.” All students in attendance were invited to participate in the study.

Intervention

This session involved the delivery of a 50-minute series of video interviews on physicians’ experiences with patient death. Seven physicians were selected to represent various specialties (Internal Medicine, Pediatrics, Neurology, Emergency Medicine, Obstetrics and Gynecology, and Psychiatry). We included two resident physicians and five attending physicians to represent various levels of clinical experience. Each participant was asked to highlight common encounters with patient death in their specialty, challenging experiences with patient death, coping strategies, and advice for medical students. Video interviews were conducted and edited by SK, the first author, for brevity and flow. The video was then presented to students during the 90-minute class session.

When students arrived in class, they were asked to complete the pre-session survey. The session then began, with students seated at tables with two to four peers. They were asked to discuss a few introductory questions related to anticipatory fears and challenges with future patient death encounters. Students then watched the first portion of the video, which described the various settings in which physicians encountered patient death, followed by a small group discussion on their initial responses to the stories physicians shared. Students then watched the second portion of the video, with interviews of various ways physicians cope with patient death. One theme physicians shared was the difficulty they still experience, even as seasoned clinicians, in coping with patient death. A frequent coping method was discussing patient death experiences with colleagues. Two physicians mentioned seeking support from a professional therapist to help cope. This was followed by a small group discussion on the coping strategies mentioned. Students also discussed which methods they anticipated using when dealing with patient death. Next, they watched several physicians describe challenging patient death situations and subsequently discussed their observations and questions raised by hearing these stories. For the final component of the session, students listened to advice provided by physician interviewees. Several physicians advised students to share their experiences with colleagues or senior clinicians. This was followed by a small group reflection on how they were impacted by the overall session.

Data collection and analysis

We utilized two previously validated scales for this study. To assess student anticipatory fears associated with patient death, we utilized the Thanatophobia Scale [8,9], which consists of seven Likert-style questions that assess student fears associated with caring for dying patients or experiences with patient death. To assess students’ perception of their ability to cope with patient death, we utilized a nine-item Likert-style scale called Bugen’s Coping With Death Scale (Short Version), previously validated for use in two countries [10,11].

Both scales were combined into a 16-item survey administered immediately prior to and after the 90-minute class session. Students were given about three minutes to complete the survey each time it was administered.

Pre- and post-session data were tested for normality with a Shapiro-Wilk test. After normality was confirmed, data points were analyzed using a paired samples t-test in IBM SPSS Statistics, version 28 (IBM Corp., Armonk, NY) to determine whether there was a significant difference in thanatophobia or coping skills before and after the intervention.

## Results

Overall, 21 of 23 students who participated in the pre-clerkship module on patient death participated in the study, representing a participation rate of 91%. Fear towards death per the Thanatophobia Scale decreased from a mean of 23.9 before the session to 21.5 afterwards, with a mean difference of 2.33 (p = .030). Participants experienced an increase in coping self-efficacy per Bugen’s Coping With Death Scale (Short Version), from a mean of 38.3 before the session to 43.0 afterwards. The mean difference was -4.667 (p = .003) (Table 1).

**Table 1:** Paired samples t-test for Thanatophobia Scale and Bugen’s Coping With Death Scale (Short Version) scores CI, confidence interval; df, degrees of freedom. Paired samples t-tests were used to analyze the difference in pre- and post-session Thanatophobia Scale and Bugen’s Coping With Death Scale (Short Version) scores. *Statistically significant at p < 0.05.

	Mean difference	Standard deviation	Standard error of mean	Lower (95% CI of the difference)	Upper (95% CI of the difference)	t	df	One-sided p-value	Two-sided p-value
Thanatophobia Scale score	2.333	4.575	.998	.251	4.416	2.337	20	.015	.030*
Bugen’s Coping With Death Scale score	-4.667	6.288	1.372	-7.529	-1.805	-3.401	20	.001	.003*

For thanatophobia, the greatest magnitude of improvement was noted in response to the statements “dying patients make me feel uneasy” (mean difference 0.91) and “I can lessen the anxiety of those around me when the topic is death and dying” (mean difference 0.67), while thanatophobia actually increased slightly in response to the statement “I don’t look forward to being the personal physician of a dying patient” (mean difference -0.04). For coping with death, student ratings increased most for the statements “I know who to contact when death occurs” (mean difference -1.19) and “I can lessen the anxiety of those around me when the topic is death and dying” (mean difference -0.91) and decreased slightly in response to “I know how to listen to others, including the terminally ill” (mean difference 0.14).

## Discussion

Our study cohort, consisting entirely of second-year medical students, scored similarly on the pre-session Thanatophobia Scale (mean 23.8) to a previously studied cohort of second-year medical students [4], suggesting our participants were similarly prepared for patient death experiences at baseline. This cohort demonstrated statistically significant improvement in thanatophobia and self-efficacy in coping with death after the session. This suggests that our single-session intervention may be an efficient and effective way to support students in future encounters with patient death. This is particularly relevant given the increasing number of topics medical educators must consider including in an undergraduate medical education curriculum.

While this quantitative design did not explore the specific session elements that led to improvement in thanatophobia and coping with death, the following are some potential reasons. This intervention focused on storytelling. Physicians shared their personal stories, including their ongoing difficulty managing their emotions in clinical settings and beyond. This might have been a key contributor to the session’s positive impact. Prior work revealed that students perceive a discrepancy between their emotional responses and those expressed by physicians [1]. This session may have helped to narrow this perceived discrepancy and contribute to a sense of common humanity. In a similar vein, a study demonstrated improvement in Bugen’s Coping With Death Scale scores in second-year medical students after participation in a mentorship program [6]. While this single-session intervention was by no means a substitute for a mentorship program, hearing the stories of physicians may have been similarly supportive.

Peer-to-peer support may have been another reason for this session’s positive impact. Many physicians encouraged students to discuss challenging patient death experiences with colleagues. Students also engaged in peer discussions throughout the session. These elements of peer-to-peer support align with another work that indicates the majority of clinicians cope with death by discussing experiences with colleagues [12]. One medical school curriculum on patient death includes peer discussion groups to process these experiences [13]. In a study on geriatric nurses, the increased frequency of sharing experiences involving terminally ill patients was also associated with improvements in Bugen’s Coping With Death Scale scores [14].

While statistically significant, the improvements in the Thanatophobia Scale and Bugen’s Coping With Death Scale (Short Version) scores were modest. This could have been due to the intervention itself. An alternate explanation is the short interval between the pre- and post-session surveys. Further spacing of these two measurements might have led to different results. In addition, the sample size was small and may have involved a selection bias, since participants were among the minority of students who enrolled in the clerkship boot camp elective. Another limitation, as mentioned above, was our quantitative methodology. While our study revealed improvements in thanatophobia and coping with death, it did not illuminate the specific reasons for these improvements. These limitations invite further exploration. Score measurements for thanatophobia and coping with death could be obtained several months into clinical rotations. In addition, a qualitative design would likely provide insights into the specific elements of the session that were most valuable to students. Such a study, conducted months into clinical training, could also illuminate how their recollection of this session impacts them during their first experiences with patient death.

## Conclusions

Medical students struggle with navigating patient death and the emotions that surround it. While this highlights a need for training and support in this area, medical educators must also consider this in the context of competing curricular elements. Our brief single-session intervention showed modest statistically significant improvements in Thanatophobia Scale and Bugen’s Coping With Death Scale scores, supporting the value of physician storytelling and peer discussion in mitigating student difficulty surrounding patient death. Educators wishing to promote student formation in this regard could feasibly include this intervention as a single session in a standing course or as part of an orientation to the clerkship curriculum. Future research could explore student narratives of the impact of physician storytelling, including student reflections after experiencing their first patient death.
